# Immune Checkpoint Inhibitors in Non-Small Cell Lung Cancer (NSCLC) Treatment: Quo Vadis?

**DOI:** 10.3390/ijms25126309

**Published:** 2024-06-07

**Authors:** Antonios N. Gargalionis, Kostas A. Papavassiliou, Athanasios G. Papavassiliou

**Affiliations:** 1 Laboratory of Clinical Biochemistry, ‘Attikon’ University General Hospital, Medical School, National and Kapodistrian University of Athens, Chaidari, 12462 Athens, Greece; agargal@med.uoa.gr; 2 First University Department of Respiratory Medicine, ‘Sotiria’ Hospital, Medical School, National and Kapodistrian University of Athens, 11527 Athens, Greece; konpapav@med.uoa.gr; 3 Department of Biological Chemistry, Medical School, National and Kapodistrian University of Athens, 11527 Athens, Greece

Lung cancer has been established as the second most common cancer worldwide (most common cancer in men and second most common cancer in women) and as the leading cause of cancer morbidity among neoplasms [1]. Non-small cell lung cancer (NSCLC) represents approximately 80–85% of lung cancer cases, and the majority of those are diagnosed in advanced stages (IIIB/C or IV) [2]. Most of the patients also develop metastases and demonstrate disease relapse even after a complete resection of the tumor [3]. Administration of immune checkpoint inhibitors (ICIs) has positively shifted survival rates and treatment responses of the patients, thus making immunotherapy an essential pillar of NSCLC treatment [2]. Levels of programmed death-ligand 1 (PD-L1) expression remain the most important predictive biomarker associated with the patients’ response to anti-programmed cell death protein 1 (PD-1)/PD-L1 immunotherapy [4,5]. Increased tumor mutational burden (≥10 mutations per megabase) also seems to predict a positive response to immune checkpoint inhibitors, as well as the detection of specific genomic alterations [6,7]. With regard to NSCLC that is diagnosed in operable stages (IB–III), phase III clinical trials clearly show that adjuvant immunotherapy (atezolizumab or pembrolizumab) following adjuvant chemotherapy augments disease-free survival [8,9]. The Food and Drug Administration (FDA) has also approved immunotherapy (nivolumab) in the context of neoadjuvant therapy in combination with platinum-based chemotherapy for stage III NSCLC patients who can be operated on. This combination has been proven to increase event-free survival and pathological complete response in IB–IIIA NSCLC resectable cases [10]. Concerning unresectable cases of locally advanced NSCLC, patients that received anti-PD-1/PD-L1 with durvalumab immunotherapy following chemotherapy and radiotherapy exhibit higher progression-free survival (PFS). The latter monoclonal antibody has been FDA-approved for maintenance treatment in NSCLC patients of stage III who are non-resectable [11,12]. In cases of advanced disease, PD-L1 expression is the main criterion for immune checkpoint inhibitor immunotherapy. The FDA has approved pembrolizumab for first-line monotherapy in advanced NSCLC when PD-L1 expression is ≥50% since the trial has shown increased PFS for these patients [13]. Atezolizumab and cemiplimab have also been approved for first-line monotherapy of advanced NSCLC in patients who fulfill the PD-L1 criterion [14,15]. In cases that do not meet the PD-L1 expression cut-off (≤50%), the FDA has approved combinatorial first-line treatment with pembrolizumab plus platinum-based chemotherapy in non-squamous NSCLC patients since the trial has revealed increased PFS compared to the standard first-line treatment control group [16]. Furthermore, the combination of nivolumab with an anti-cytotoxic T-lymphocyte-associated protein 4 (CTLA-4), also known as CD152, inhibitor (ipilimumab), has better results than chemotherapy alone in advanced NSCLC tumors [17]. According to the clinical and pathological features of the patient, other combinatorial treatments have been suggested, including standard chemotherapy, immunotherapy, and anti-vascular endothelial growth factor-A (VEGF-A) monoclonal antibody (bevacizumab), as well as treatment with dual checkpoint inhibitors [2,18]. Immune checkpoint inhibitors have also been approved for second-line treatment in NSCLC [19].

Although immunotherapy has revolutionized treatment options in NSCLC, there are several challenges that need to be overcome to gain optimal efficacy. It has been documented that treatment with immune checkpoint inhibitors demonstrates poor efficacy in NSCLC patients with oncogene driver mutations, such as those harbored on *epidermal growth factor receptor* (*EGFR*; a receptor tyrosine kinase (RTK)) and *anaplastic lymphoma kinase* (*ALK*; also known as ALK tyrosine kinase receptor or CD246) genes. Therefore, there is an unmet need to elucidate mechanisms of resistance and combine immunotherapy with tyrosine kinase inhibitors (TKIs) in certain NSCLC subgroups [18]. Alternative approaches also suggest the therapeutic potential of inhibitors against new immune checkpoint targets and novel combinatorial treatments of immune checkpoint inhibitors with cytokines, stimulator of interferon genes (STING; an endoplasmic reticulum-associated membrane protein that plays an essential role in the innate immune response) agonists, and adenosine antagonists (e.g., A1 adenosine receptor (A1AR) antagonists that increase caspase 3 and 7 activity in lung cancer cells) [18]. There is also a need to provide more reliable biomarkers for treatment-related decisions, but also to improve accuracy and standardize methods for the biomarkers that are already in use. PD-L1 expression is an established biomarker that guides immunotherapy-related treatment; however, there are inherent weaknesses regarding immunohistochemical evaluations of P-L1 expression that partially explain differences in immunotherapy responses between different clinical trials [20]. Further improvement with more accurate detection of predictive biomarkers and established criteria for patient selection will enhance therapeutic efficacy; hence, there will be more patients that could benefit from immunotherapy and gain prolonged survival.
